# Attitudes toward an HPV vaccine for condyloma acuminata and willingness to undergo vaccination among STD clinic attendees in China: Focus on STI prevention with HPV vaccine

**DOI:** 10.1186/s12889-024-18904-0

**Published:** 2024-06-17

**Authors:** Anqi-Liu, Jiali-Quan, Lingxian-Qiu, Yue-Huang, Wujian-Ke, Huachun-Zou, Ting-Wu, Xuqi-Ren

**Affiliations:** 1 Department of Dermatology, Affiliated Hospital of Nantong University, Nantong, China; 2 State Key Laboratory of Vaccines for Infectious Diseases, Xiang An Biomedicine Laboratory, Department of Laboratory Medicine, School of Public Health, Xiamen University, Xiamen, China; 3 National Institute of Diagnostics and Vaccine Development in Infectious Diseases, State Key Laboratory of Molecular Vaccinology and Molecular Diagnostics, National Innovation Platform for Industry-Education Integration in Vaccine Research, NMPA Key Laboratory for Research and Evaluation of Infectious Disease Diagnostic Technology, Xiamen University, Xiamen, China; 4 Department of Sexually Transmitted Diseases, Dermatology Hospital, Southern Medical University, Guangzhou, China; 5 School of Public Health, Fudan University, Shanghai, China

**Keywords:** Condyloma acuminata, HPV, Vaccine, STD clinic attendees, Acceptance, Knowledge

## Abstract

**Background:**

Condyloma acuminata (CA) is a common, and recurrent sexually transmitted disease (STD) that greatly contributes to direct health care costs and has a substantial psychosocial impact. Human papillomavirus (HPV) vaccination (containing L1 protein for HPV types 6 and 11) effectively controls CA.

**Objectives:**

We investigated attitudes toward the HPV vaccine for CA and willingness to undergo vaccination among STD clinic attendees in China.

**Methods:**

Attendees at STD clinics at two selected hospitals in Guangdong and Jiangsu Provinces from May to September 2017 were requested to complete a self-administered questionnaire for this cross-sectional study.

**Results:**

The participants’ median age was 28 years (IQR: 24.0–34.0), and the sex ratio was balanced; 63.5% were from Guangdong, 36.5% were from Jiangsu, and 44.5% had a history of CA. The vaccine acceptance rate was high among the participants (85.8%,235/274) to whom the HPV vaccine for CA was available, especially among those who had heard of CA (89.0%, AOR = 3.14, 95% CI: 1.29–7.63, *p =* 0.0114). 95 (34.7%) of 274 participants had a positive attitude toward the HPV vaccine for CA. STD clinic attendees who had heard of the connection between HPV and CA (AOR = 2.56, 95% CI: 1.31-5.00, *p =* 0.0060), had heard of the HPV vaccines or cervical cancer vaccines (AOR = 1.90, 95% CI: 1.02–3.54, *p =* 0.0444) and had ever proactively discussed CA or the vaccine with others (AOR = 1.95, 95% CI:1.00-3.79, *p =* 0.0488) had better attitudes toward the HPV vaccine for CA. Over half of the participants (52.5%) expected the price of the HPV vaccine for CA to be under $90.

**Conclusion:**

The acceptance of the HPV vaccine for CA was high among STD clinic attendees in China, and the participants’ self-perceived knowledge of CA and HPV was associated with better attitudes toward the HPV vaccine for CA. Education to improve knowledge is vital for reducing vaccine hesitancy.

## Introduction

Human papillomavirus (HPV) is known to be associated with various cancers, especially cervical cancer. In 2018, 13% of cancer cases worldwide were attributable to infection, and HPV infection accounted for 31% of all infection-related incident cancer cases [1]. Cervical cancer is the most common HPV-attributable cancer, accounting for 80%; over 95% of cervical cancer cases from HPV infection [2]. China is one of the countries in great part responsible for the cases, with approximately 0.11 million cases [2]. ​Therefore, many people in China associate HPV with cervical cancer, however, HPV also causes condyloma acuminata (CA, also known as anogenital warts or genital warts). Despite cancer being a priority, we are also concerned about the neglected disease burden caused by CA [3]. Notably, CA is potentially an important co-factor in HIV transmission [4, 5].

CA is a frequently identified clinical symptom of low-risk HPV infection. HPV type 6 (HPV-6) and HPV-11 are associated with approximately 90% of CA cases [6]. The reported incidence of CA in China (0.24–0.29/1000 person years) is lower than that in the general population worldwide (1.6–2.9/1000 person years). However, the true disease burden associated with CA in China, is seriously underestimated due to incomplete disease surveillance and registration systems [7–9]. Furthermore, CA often recurs after initial remission is achieved and requires repeated treatments, with recurrence rates ranging from 6–77% [10]. Although CA is the most frequent benign lesion and is not life-threatening, it is a common problem with a considerable impact on health-care costs; additionally, CA is a source of tremendous psychological pressure and declining patient quality of life [3, 11, 12]. CA is worthy of further attention in high-risk STD populations.

Vaccination has become an effective preventive tool for precancerous lesions and CA. As of September 2023, 64% WHO member states have incorporated the HPV vaccine into their national immunization schedules. Predominantly, these are high-income and upper-middle-income countries; however, many of the world’s most populous nations have not yet included the HPV vaccine in their immunization regimes, with only 12% of females and 6% of males having received the first dose as of 2022 [13]. Despite the increased introduction of the HPV vaccine in low-income and lower-middle-income countries (LLMICs) with the support of Gavi, a persistent global shortage of the HPV vaccine has led to concerns that supply may not meet the increasing worldwide demand. Furthermore, while some nations have embraced a gender-neutral vaccination policy, targeting both boys and girls, the LLMICs primary emphasis continues to be on vaccinating girls aged 9–10 years, a practice that contrasts with the broader age range targeted in developed countries.

HPV vaccines were introduced in mainland China in 2016 [14]. A bivalent vaccine (2vHPV, Cervarix®), and a quadrivalent vaccine (4vHPV, Gardasil^®^), were approved in July 2016 and April 2017, respectively. A 9-valent HPV vaccine (9vHPV, Gardasil^®^9) was licensed in May 2018. The domestic bivalent Innovax’s (Cecolin^®^) and Walvax’s HPV vaccines (Walrinvax^®^) were approved in December 2019 and March 2022, respectively. Each of the five vaccines is licensed for different age ranges at initial, with Cervarix^®^ recommended for ages of 9–25, Gardasil^®^ recommended for ages 20–45, Gardasil^®^9 recommended for ages 16–26, and the two domestic bivalent HPV vaccines are recommended for individuals aged 9–45 and 9–30, respectively. While currently all the HPV vaccines have been proved safe for use in women aged 9–45 years in China (excluding Walrinvax^®^) and have not yet been included in the National Immunization Programme [15]. Immunization of women aged 9–26 is the priority in Chinese guidelines. The current supply of HPV vaccine is insufficient to cover the target population to prevent cancer; thus, there are insufficient vaccines available for the implementation of vaccination to address CA, especially the quadrivalent (Gardasil^®^) and the nonvalent (Gardasil^®^9) HPV vaccines, the only two licensed HPV vaccines that protect against CA [14]. The unaffordable price ($370–$595) has largely limited vaccine uptake by individuals in high-risk populations. Hence, an affordable HPV-6/11 vaccine for preventing CA will be applicable to the population at high risk for STDs and may be a potential method to improve the disease burden associated with CA in the future.

At present, one HPV 6/11 bivalent vaccine candidate has specifically been designed to protect against genital warts and other HPV 6/11-related diseases. This vaccine has been preliminarily proven to be well tolerated and demonstrated robust immunogenicity in a phase 1 clinical study (NCT02405520) and is currently being evaluated in a phase 2 clinical trial (NCT02710851) [16]. Despite being in the early stages of validation, the introduction of such a vaccine could potentially contribute to a lower prevalence of CA in high-risk sexually transmitted infections (STIs) populations. The question arises, however, we are interested in exploring whether a vaccine specifically for the prevention of CA is needed when there is already an HPV vaccine for the prevention of both cancer and CA, and whether STD clinic attendees (who are at high risk for STIs, but have a lower chance of receiving quadrivalent or nonvalent HPV vaccines) will be the target potential population? In general, the purpose of this study was to investigate the attitudes toward HPV vaccination for CA and the willingness to undergo vaccination among STD clinic attendees in China.

## Materials and methods

### Design and participants

A cross-sectional survey covering the knowledge of, attitude toward, and willingness to undergo the HPV vaccine for CA was conducted at the Dermatology Hospital of Southern Medical University and the Affiliated Hospital of Nantong University from May to September 2017. Both centers were visited by people with STIs requiring treatment or who suspected they had an STI. We recruited all individuals who visited the two STD clinics from May to September 2017. STD clinic attendees aged 18 years or older were consecutively included after consent and were asked to complete a self-administered questionnaire designed by a research team comprising physicians and public health and sociology experts, and revised after a pre-survey evaluation in January 2017.

### Data collection and questionnaire

The questionnaire covering knowledge, attitude, and practices (KAP) regarding CA and HPV vaccines/cervical cancer vaccines, included information on sociodemographic characteristics, sexual behaviors, history of infection with HPV, HIV and syphilis, the individual’s self-perception on their knowledge of CA and the HPV vaccines/cervical cancer vaccines, and the acceptance of the HPV vaccine for CA and their behavioral willingness regarding vaccination.

The study protocol was reviewed and approved by the Ethics Committees of Guangdong Provincial Dermatology Hospital, Guangzhou, China on November 21, 2016 (approval number: GDDHLS-2016112102). After informed consent was obtained, all eligible participants completed the paper questionnaire independently in a private consulting room, and assistance was provided if required. The questionnaire took approximately 10 min to complete. The collected data were input separately by two different researchers and checked by a third researcher to ensure accuracy.

### Statistical analysis

Descriptive statistics were used to compile the participants’ demographic characteristics and their sexual behaviors, history of STIs, and knowledge of CA and HPV vaccines (cervical cancer vaccines) and attitude toward the HPV vaccine for CA. A chi-square test was used to compare categorical variables and test the differences between males and females based on these items. The crude odds ratio (OR) was first proposed in a univariate analysis. The univariate logistic regression analysis began with a full set of sociodemographic, sexual behaviors, history of STIs, knowledge of CA and HPV vaccines /cervical cancer vaccines, and attitude toward the HPV vaccine for CA, and behavioral willingness regarding vaccination to evaluate the associations with the willingness to undergo vaccination and attitudes toward the HPV vaccine for CA. Other variables with a P value < 0.05 and a dummy variable P value < 0.2 in the univariate models were entered into a multivariate logistic regression model to explore factors associated with the HPV vaccine for CA acceptance. The adjusted odds ratio (AOR) and the corresponding 95% confidence intervals (CI) were calculated to assess the results of the regression model. Data regarding the average costs per visit were normally distributed, and the results were analyzed using Student’s t test; otherwise, the rank sum test was used. Statistical analyses were conducted using SAS 9.4, with the significance threshold of a P value of 0.05.

Attitude toward the HPV vaccine for CA: The attitude section of the questionnaire included 3 questions on the individual’s view on the HPV vaccine for CA safety and effectiveness and their willingness to recommend vaccination; the questions were on a six-point scale for assessing individual attitudes (5 points - Strongly Agree, 4 points - Agree, 3 points - Neutral, 2 points - Disagree, 1 point - Strongly Disagree, 0 points - Unclear). Participants were considered to have a positive attitude toward the HPV vaccine for CA when they scored more than 9 points on the 3 attitude questions.

## Result

### Demographic characteristics, sexual behaviors, and history of STIs

After excluding 40 incomplete questionnaires, 274 (87.3%) participants were included in the study, among whom 174(63.5%) were from Guangdong and 100(36.5%) were from Jiangsu. The detailed baseline information of the included participants was summarized in Table 1, including the sociodemographic characteristics, sexual behaviors, and history of STIs. The participants were distributed evenly by sex (55.5% vs. 44.5%), and the median age was 28 years old (IQR: 24.0–34.0). Furthermore, 88.0% of participants were heterosexual. Approximately half of the participants were married (55.8%), while 80.4% had fixed sexual partners over the past 12 months. The prevalence of CA among the STD clinic attendees was 44.5% (122/274). In 122 STD clinic attendees with a history of CA, 69.7% (85/122) currently had CA, and 56.6% (69/122) had a history of recurrent CA, with an interval of 1–3 months (65.7%) in about two-thirds of them. Furthermore, 11.0% of participants self-reported that they were living with HIV, and 4.7% had been previously diagnosed with syphilis.

**Table 1:** Sociodemographic characteristics, sexual behaviors, and STIs history in STD clinic attendees

Variable	Sex		Total	χ^2^	*P*
	Female%	Male%			
**Sociodemographic characteristics**					
Site					
Guangzhou	104(68.4)	70(57.4)	174(63.5)	3.5617	0.0591
Jiangsu	48(31.6)	52(42.6)	100(36.5)		
Age					
18 ~ 25 years	57(37.5)	39(32.0)	96(35.0)	1.9567	0.3759
26 ~ 40 years	85(55.9)	70(57.4)	155(56.6)		
> 40 years	10(6.6)	13(10.7)	23(8.4)		
Educational attainment					
high school or below	62(40.8)	49(40.2)	111(40.5)	0.1283	0.9379
Bachelor degree	85(55.9)	68(55.7)	153(55.8)		
Master degree and higher	5(3.3)	5(4.1)	10(3.3)		
Marital status					
Single	51(33.6)	53(43.4)	104(38.0)	2.8137	0.2449
Married	91(59.9)	62(50.8)	153(55.8)		
Separation / Divorce	10(6.6)	7(5.7)	17(6.2)		
Monthly salary (RMB)^*^					
≦ 2,000	26(17.7)	11(9.2)	37(13.9)	10.9609	0.0119
2,000–4,999	67(45.6)	44(36.7)	111(41.6)		
5,000–9,999	32(21.8)	46(38.3)	78(29.2)		
≧ 10,000	22(15.0)	19(15.8)	41(15.4)		
**Different sexual behaviors**					
Self-identified sexual orientation					
MSM	0(0.0)	28(23.0)	28(10.2)	/	< 0.0001
Heterosexual	151(99.3)	90(73.8)	241(88.0)		
Bisexual	0(0.0)	0(0.0)	0(0.0)		
Unsure	1(0.7)	4(3.3)	5(1.8)		
Fixed sexual partners in the past 12 months^*^					
Yes	133(88.7)	84(70.0)	217(80.4)	14.7243	0.0001
No	17(11.3)	36(30.0)	53(19.6)		
Temporary sexual partners in the past 12 months^*^					
Yes	31(22.5)	53(44.9)	84(32.8)	14.5440	0.0001
No	107(77.5)	65(55.1)	172(67.2)		
Number of sexual partners in the past 12 months^*^					
<5	91(68.4)	85(72.7)	176(70.4)	11.3759	0.0034
≧ 5	2(1.5)	11(9.4)	13(5.2)		
0	40(30.1)	21(18.0)	61(24.4)		
Frequency of condom use during sex with fixed partner in the past 12 months^*¶^					
Every time	24(20.3)	24(21.9)	40(20.9)	2.6880	0.4423
Always	23(19.5)	21(28.8)	44(23.0)		
Sometimes	53(44.9)	27(37.0)	80(41.9)		
Never	18(15.2)	9(12.2)	27(14.1)		
Frequency of condom use during sex with temporary partner in the past 12 months ^*ξ^					
Every time	4(17.4)	20(40.0)	24(32.9)	4.8581	0.1825
Always	6(26.1)	14(28.0)	20(27.4)		
Sometimes	8(34.8)	10(20.0)	18(24.7)		
Never	5(21.7)	6(12.0)	11(15.1)		
Addictive drugs or substances in the past 12 months^*^					
Yes	1(0.7)	2(1.7)	3(1.1)	/	0.5925
No	143(99.3)	118(98.3)	261(98.9)		
Sexual intercourse under the influence of alcohol in the past 12 months^*^					
Yes	19(12.6)	32(26.7)	51(18.8)	8.6812	0.0032
No	132(87.4)	88(73.3)	220(81.2)		
Frequency of drinking in the past 12 months^*^					
Constantly	7(4.6)	24(19.8)	31(11.4)	23.3827	< 0.0001
Sometimes	68(45.0)	64(52.9)	132(48.5)		
No drinking	76(50.3)	33(27.3)	109(40.1)		
Frequency of smoking in the past 12 months^*^					
No smoking	144(95.4)	66(54.6)	210(77.2)	65.4037	< 0.0001
1–5 per day	5(3.3)	17(14.1)	22(8.1)		
Over 5 per day	2(1.3)	38(31.4)	40(14.7)		
**History of STIs**					
History of CA					
Yes	60(39.5)	62(50.8)	122(44.5)	3.5272	0.0604
No	92(60.5)	60(49.2)	152(55.5)		
How many times did you have CA in the past 1 year^*δ^					
0	13(25.0)	7(11.7)	20(17.9)	5.2989	0.1512
1	5(9.6)	6(10.0)	11(9.8)		
2	29(55.8)	34(56.7)	63(56.3)		
≧ 3	5(9.6)	13(21.7)	18(16.1)		
Current status of CA^*^^δ^					
Yes	40(70.2)	45(75.0)	85(72.7)	0.3424	0.5584
No	17(29.8)	15(25.0)	32(27.4)		
Recurrent history of CA^*δ^					
Yes	31(60.8)	38(65.5)	69(63.3)	0.2617	0.6090
No	20(39.2)	20(34.5)	40(36.7)		
Interval of recurrence^*§^					
1-3months	20(69.0)	24(63.2)	44(65.7)	/	0.1709
4-6months	2(6.9)	4(10.5)	6(9.0)		
7-9months	1(3.5)	2(5.3)	3(4.5)		
10-12months	0(0.0)	5(13.2)	5(7.5)		
others	6(20.7)	3(7.9)	9(13.4)		
HIV testing status^*^					
Have never tested before	46(35.4)	28(26.4)	74(31.4)	2.2254	0.3287
HIV-negative	71(54.6)	65(61.3)	136(57.6)		
Living with HIV	13(10.0)	13(12.3)	26(11.0)		
History of syphilis ^*^					
Have never tested before	54(42.5)	36(34.0)	90(38.6)	6.9761	0.0306
No	71(56.0)	61(57.6)	132(56.7)		
Yes	2(1.6)	9(8.5)	11(4.7)		

*Data partially missing

^¶^Statistics were calculated based on 217 STD clinic attendees who had fixed sexual partners in the past 12 months

^ξ^Statistics were calculated based on 84 STD clinic attendees who had temporary sexual partners in the past 12 months

^δ^Statistics were calculated based on 122 STD clinic attendees who had a history of CA

^§^Statistics were calculated based on 69 STD clinic attendees who had a recurrent history of CA

Among the participants, males had a higher salary than females (*χ2* = 10.9609, *p =* 0.0119). In addition, male STD clinic attendees were more likely than female STD clinic attendees to have engaged in risky sexual behaviors in the past 12 months, such as having temporary sexual partners (*χ2* = 14.5440, *p* = 0.0001), having over 5 sexual partners (*χ2* = 11.3759, *p* = 0.0034), and participating in sexual intercourse under the influence of alcohol (*χ2* = 8.6812, *p* = 0.0032). Additionally, male STD clinic attendees were more likely than females to engage in unhealthy life behaviors, such as frequent drinking (*χ*2 = 23.3827, *p* < 0.0001) and smoking (*χ* 2 = 65.4037, *p* < 0.0001). Compared with female STD clinic attendees, male STD clinic attendees were more likely to have been diagnosed with syphilis. (*χ*2 = 6.9761, *p* = 0.0306). There were no significant sex-based differences in self-reported HIV testing status or history of CA.

### Self-perception on knowledge of CA and HPV vaccines

**Fig. 1:**
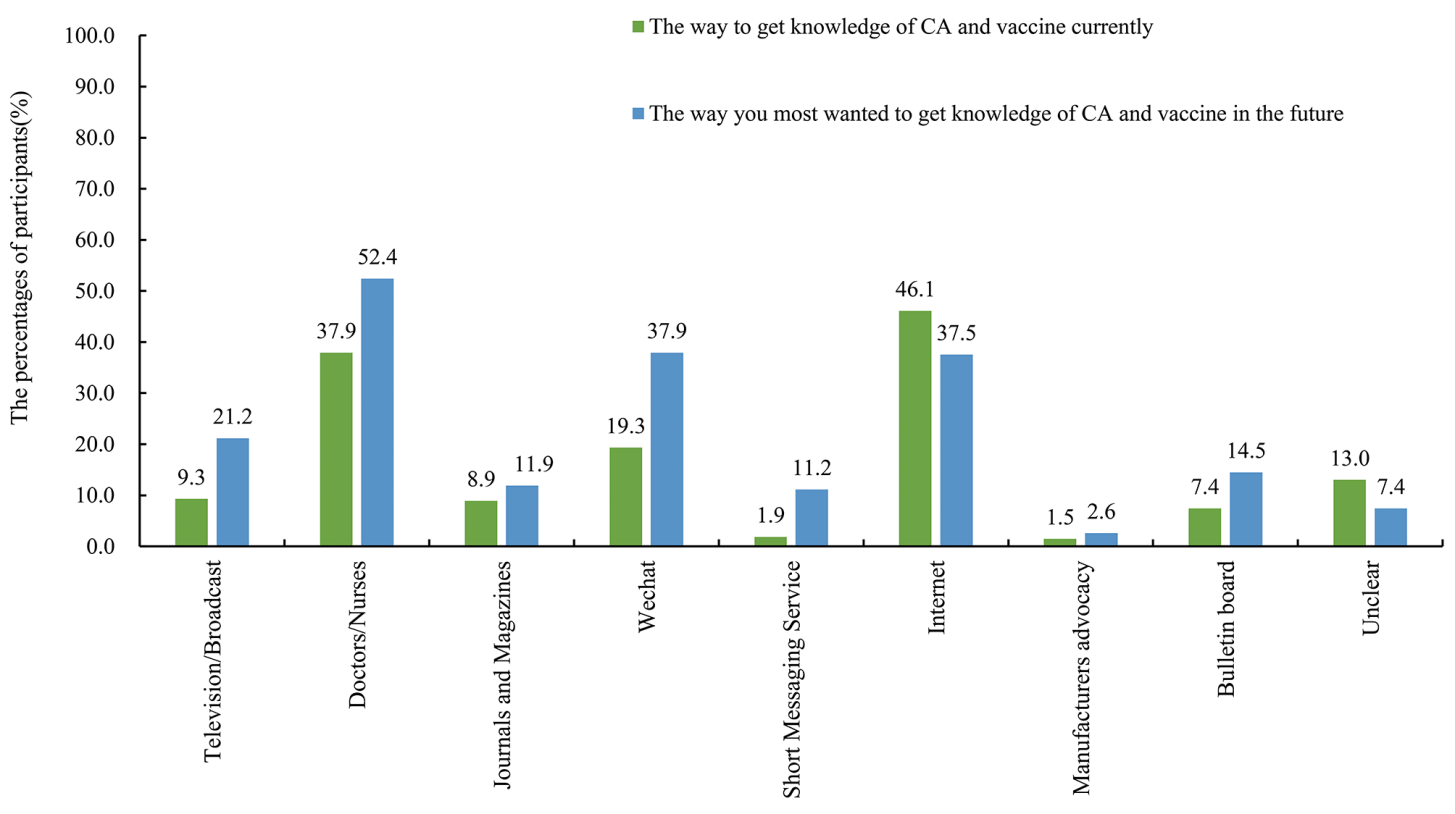
The sources of CA and vaccine knowledge. The x axis listed difference sources of knowledge. The y axis represents the percentages of participants getting knowledge with these sources. Participants were allowed to select multiple options when answering questions about their current sources of knowledge on CA and vaccines and their preferred future sources of information on the same topics

Table 2 showed the participants’ self-perception on their knowledge of CA and HPV vaccines. Approximately 72.3% of STD clinic attendees knew about CA and 42.0% of them had heard about the HPV vaccines /cervical cancer vaccines, with the internet being the main source of this information (around 46.1%), followed by the doctors (37.9%), WeChat (19.3%), and television/broadcast (9.3%) (Fig. 1). However, a sizable proportion of CA disease-aware STD clinic attendees still had poor knowledge of the disease; 39.4–52.2% of STD clinic attendees lacked knowledge of the transmission routes, symptoms and risks associated with CA or of the treatments available for CA.

**Table 2:** Self-perception on knowledge of CA and vaccines in STD clinic attendees

Variable	Sex		Total	χ^2^	*P*
	Female%	Male%			
**Self-perception on knowledge of CA**					
Ever heard of CA					
Yes	103(67.8)	95(77.9)	198(72.3)	3.4483	0.0633
No	49(32.2)	27(22.1)	76(27.7)		
Know the route of transmission of CA^ξ^					
Yes	62(60.2)	58(61.0)	120(60.6)	0.0153	0.9017
No	41(39.8)	37(39.0)	78(39.4)		
Know the symptoms of CA^ξ^					
Yes	60(58.3)	54(56.8)	114(57.6)	0.0402	0.8410
No	43(41.8)	41(43.2)	84(42.4)		
Know the risks of CA^ξ^					
Yes	54(52.4)	52(54.7)	106(53.5)	0.1060	0.7448
No	49(47.6)	43(45.3)	92(46.5)		
Know the treatments of CA^ξ^					
Yes	49(47.6)	45(47.4)	94(47.5)	0.0008	0.9770
No	54(52.4)	50(52.6)	104(52.5)		
Ever heard of HPV infection*					
Yes	101(69.7)	78(65.6)	179(67.8)	0.5055	0.4771
No	44(30.3)	41(34.4)	85(32.2)		
Ever heard of the connection between HPV and CA*					
Yes	85(59.0)	72(61.0)	157(59.9)	0.1069	0.7438
No	59(41.0)	46(39.0)	105(40.1)		
**Self-perception on knowledge of HPV vaccines**					
Ever heard of HPV vaccine /cervical cancer vaccine					
Yes	75(49.3)	40(32.8)	115(42.0)	7.6160	0.0058
No	77(50.7)	82(67.2)	159(58.0)		
HPV vaccine can prevent CA^*^^¶^					
Yes	44(59.5)	29(72.5)	73(64.0)	1.9173	0.1662
No	30(40.5)	11(27.5)	41(36.0)		
Know the vaccination procedure^*¶^					
Yes	26(35.1)	10(25.0)	36(31.6)	1.2344	0.2666
No	48(64.9)	30(75.0)	78(68.4)		
**Behavioral willingness for vaccination**					
Have any of your friends or family members received the HPV vaccines^*^					
Yes	29(21.5)	17(14.9)	46(18.5)	1.7710	0.1833
No	106(78.5)	97(85.1)	203(81.5)		
Have you proactively learned about CA^*^					
Yes	65(48.2)	52(44.4)	117(48.4)	0.3457	0.5566
No	70(51.8)	65(55.6)	135(53.6)		
Have you proactively learned about HPV vaccine^*^					
Yes	44(32.6)	33(28.2)	77(30.6)	0.5686	0.4508
No	91(67.4)	84(71.8)	175(69.4)		
Have you received education about CA^*^					
Yes	28(20.7)	25 (21.4)	53(21.0)	0.0148	0.9031
No	107(79.3)	92 (78.6)	199(79.0)		
Have you proactively discussed CA or the vaccine with others^*^					
Yes	32(23.7)	28(23.9)	60(23.8)	0.0018	0.9662
No	103(76.3)	89(76.1)	192(76.2)		

* Data partially missing

^ξ^Statistics were calculated based on 198 STD clinic attendees who had heard of CA

^¶^Statistics were calculated based on 115 STD clinic attendees who had heard of the HPV vaccine

Although 67.8% of them had heard of HPV infection, 40.1% were unaware of the connection between CA and HPV. STD clinic attendees seemingly had poor behavioral willingness toward the HPV vaccine; only 18.5% of the STD clinic attendees evaluated in this study had friends or family members who had received the HPV vaccines/cervical cancer vaccines, and 21.0–30.6% had proactively obtained knowledge regarding HPV vaccination, receiving related education or discussing with others.

Among the participants, female STD clinic attendees were more likely than male STD clinic attendees to hear of HPV vaccines/cervical cancer vaccines (*χ*2 = 7.6160, *p* = 0.0058). In addition, there was no significant difference between males and females in regards to knowledge of CA and HPV vaccines/cervical cancer vaccines and behavioral willingness toward vaccination.

### Factors associated attitudes toward the HPV vaccine for CA and willingness to undergo vaccination

A high willingness to undergo the HPV vaccine for CA was observed (85.8%, 235/274), in our study, and no sex differences were present (male vs. female: 87.7% vs.84.2%). However, only 95 (34.7%) of 274 participants had a positive attitude toward the HPV vaccine for CA. Tables 3 and 4 showed the factors associated with attitudes toward the HPV vaccine for CA and willingness to undergo vaccination for CA. The univariate logistic regression analysis revealed that, among STD clinic attendees, the 9 variables “Age”, “Self-identified sexual orientation”, “Sexual intercourse under the influence of alcohol in the past 12 months”, “History of CA”, “HIV testing status”, “Ever heard of CA”, “Ever heard of HPV infection”, “Ever heard of HPV vaccines/cervical cancer vaccine”, and “Have you proactively learned about CA” were later evaluated in the multivariate logistic regression analysis to predict STD clinic attendees’ willingness to receive the HPV vaccine for CA. A similar procedure was also applied to attitudes toward the HPV vaccine for CA among STD clinic attendees.

**Table 3:** Willingness to undergo CA vaccination and attitudes toward CA vaccination in STD clinic attendees with different characteristics

Variable	Willingness to CA vaccination	Crude odds ratio		Positive attitude to CA vaccination	Crude odds ratio	
	*n* (%)*	OR (95%CI)	*P*	*n* (%)^#^	OR (95%CI)	*P*
**Sociodemographic characteristics**						
Sex						
Male	107(87.7)	1.33(0.67,2.68)	0.4117	44(36.1)	1.12(0.68,1.84)	0.6641
Female	128(84.2)	ref		51(33.6)	ref	
Site						
Guangzhou	152(87.4)	1.42(0.71,2.81)	0.3219	73(42.0)	2.56(1.46,4.49)	0.0010
Jiangsu	83(83.0)	ref		22(22.0)	ref	
Age						
18 ~ 25 years	89(92.7)	5.56(1.72,18.01)	0.0042	43(44.8)	1.85(0.70,4.92)	0.2144
26 ~ 40 years	130(83.9)	2.28(0.85,6.10)	0.1022	45(29.0)	0.94(0.36,2.43)	0.8903
> 40 years	16(69.6)	ref		7(30.4)	ref	
Educational attainment						
high school or below	92(82.9)	ref		26(23.4)	ref	
Bachelor degree	134(87.6)	1.46(0.73,2.90)	0.2848	65(42.5)	2.42(1.40,4.16)	0.0015
Master degree and higher	9(90.0)	1.86(0.22,15.55)	0.5674	4(40.0)	2.18(0.57,8.32)	0.2542
Marital status						
Single	94(90.4)	ref		48(46.2)	ref	
Married	126(82.4)	0.50(0.23,1.08)	0.0759	42(27.5)	0.44(0.26,0.75)	0.0022
Separation/Divorce	15(88.2)	0.80(0.16,4.01)	0.7838	5(29.4)	0.49(0.16,1.48)	0.2037
Monthly salary (RMB)						
≦ 2,000	33(89.2)	ref		13(35.1)	ref	
2,000–4,999	91(82.0)	0.55(0.18,1.73)	0.3084	29(26.1)	0.65(0.29,1.45)	0.2945
5,000–9,999	68(87.2)	0.82(0.24,2.83)	0.7584	30(38.5)	1.15(0.51,2.61)	0.7306
≧ 10,000	37(90.2)	1.12(0.26,4.84)	0.8782	18(43.9)	1.45(0.58,3.61)	0.4302
**Different sexual behaviors**						
Self-identified sexual orientation						
MSM	27(96.4)	4.74(0.63,36.00)	0.1324	15(53.6)	2.41(1.09,5.31)	0.0291
Heterosexual	205(85.1)	ref		78(32.4)	ref	
Unsure	3(60.0)	0.26(0.04,1.63)	0.1517	2(40.0)	1.39(0.23,8.51)	0.7195
Fixed sexual partners in the past 12 months						
Yes	188(86.6)	1.33(0.59,3.00)	0.4981	73(44.6)	0.71(0.39,1.32)	0.2834
No	44(83.0)	ref		22(41.5)	ref	
Temporary sexual partners in the past 12 months						
Yes	75(89.3)	1.48(0.66,3.33)	0.3383	36(42.9)	1.64(0.96,2.81)	0.0724
No	146(84.9)	ref		54(31.4)	ref	
Numbers of sexual partners in the past 12 months						
<5	156(88.6)	2.11(0.98,4.56)	0.0568	68(38.6)	1.50(0.80,2.82)	0.2030
≧ 5	10(76.9)	0.90(0.22,3.77)	0.8884	5(38.5)	1.49(0.43,5.19)	0.5282
0	48(78.7)	ref		18(29.5)	ref	
Addictive drugs or substances in the past 12 months						
Yes	2(66.7)	ref		1(33.3)	ref	
No	225(86.2)	3.13(0.28,35.36)	0.3573	90(34.5)	1.05(0.09,11.76)	0.9670
Sexual intercourse under the influence of alcohol in the past 12 months						
Yes	49(96.1)	4.95(1.15,21.27)	0.0314	23(45.1)	1.72(0.93,3.20)	0.0850
No	183(83.2)	ref		71(32.3)	ref	
Frequency of drinking in the past 12 months						
Constantly	26(81.3)	1.09(0.53,2.25)	0.8170	13(41.9)	1.59(0.70,3.62)	0.2659
Sometimes	114(86.4)	0.90(0.30,2.67)	0.8419	47(35.6)	1.22(0.71,2.09)	0.4706
No drinking	93(85.3)	ref		34(31.2)	ref	
Frequency of smoking in the past 12 months						
No smoking	179(85.2)	ref		71(33.8)	ref	
1–5 per day	18(81.8)	0.78(0.25,2.46)	0.6705	10(45.5)	1.63(0.67,3.96)	0.2792
Over 5 per day	36(90.0)	1.56(0.52,4.69)	0.4295	14(35.0)	1.05(0.52,2.14)	0.8842
**History of STIs**						
History of CA						
Yes	111(91.0)	2.28(1.08,4.79)	0.0298	48(39.3)	1.45(0.88,2.39)	0.1461
No	124(81.6)	ref		47(30.9)	ref	
HIV testing status						
Have never tested before	60(64.6)	ref		20(27.0)	ref	
HIV-negative	122(89.7)	2.03(0.91,4.54)	0.0831	54(39.7)	1.78(0.96,3.30)	0.0678
Living with HIV	24(92.3)	2.80(0.59,13.26)	0.1945	14(53.9)	3.15(1.25,7.95)	0.0152
History of syphilis ^¶^						
Have never tested before	75(83.3)	ref		29(32.2)	ref	
No	119(90.2)	1.81(0.83, 4.00)	0.1374	51(38.6)	1.32(0.75,2.33)	0.3290
Yes	11(100.0)	4.72(0.23, 95.56)	0.3118	6(54.6)	2.52(0.71,8.96)	0.1519

^¶^Firth penalized maximum likelihood estimation (willingness to CA vaccination)

*n (%):number of those willing to receive CA vaccination (the number of those willing to receive CA vaccination/ the total number of individuals in each category)

^#^n (%): number of those having positive attitude to CA vaccination (number of those having positive attitude to CA vaccination / the total number of individuals in each category)

**Table 4:** Vaccination willingness and attitudes of STD clinic attendees with different awareness levels of CA and vaccines

Variable	Willingness to CA vaccination	Crude odds ratio		Positive attitude to CA vaccination	Crude odds ratio	
	*n* (%)*	OR (95%CI)	*P*	*n* (%)^#^	OR (95%CI)	*P*
**self-perception on knowledge of CA**						
Ever heard of CA						
Yes	178(89.0)	2.97(1.48,5.95)	0.0022	74(37.4)	1.56(0.88,2.79)	0.1309
No	57(75.0)	ref		21(27.6)	ref	
Ever heard of HPV infection						
Yes	162(90.5)	2.74(1.34,5.60)	0.0056	74(41.3)	2.29(1.28,4.10)	0.0053
No	66(77.7)	ref		20(23.5)	ref	
Ever heard of the connection between HPV and CA						
Yes	140(89.2)	1.82(0.90,3.69)	0.0972	70(44.6)	2.87(1.64,5.02)	0.0002
No	86(81.9)	ref		23(21.9)	ref	
**self-perception on knowledge of HPV vaccines**						
Ever heard of HPV vaccine/cervical cancer vaccine						
Yes	107(93.0)	3.24(1.43,7.34)	0.0049	57(49.6)	3.13(1.87,5.24)	< 0.0001
No	128(80.5)	ref		38(23.9)	ref	
**Behavioral willingness for vaccination**						
Have any of your friends or family members received the HPV vaccines						
Yes	44(95.7)	3.80(0.88,16.58)	0.0740	23(50.0)	2.38(1.24,4.57)	0.0090
No	173(85.2)	ref		60(29.6)	ref	
Have you proactively learned about CA						
Yes	109(93.2)	3.10(1.34,7.17)	0.0083	50(42.7)	2.22(1.30,3.78)	0.0035
No	110(81.5)	ref		34(25.2)	ref	
Have you proactively learned about HPV vaccine						
Yes	71(92.2)	2.16(0.85,5.47)	0.1044	39(50.7)	2.96(1.69,5.19)	0.0001
No	148(84.6)	ref		45(25.7)	ref	
Have you received education about CA						
Yes	49(92.5)	2.09(0.70,6.23)	0.1861	24(45.3)	1.92(1.03,3.56)	0.0396
No	170(85.4)	ref		60(30.2)	ref	
Have you proactively discussed CA or the vaccine with others						
Yes	56(93.3)	2.49(0.84,7.40)	0.1004	31(51.7)	2.8(1.54,5.09)	0.0007
No	163(84.9)	ref		53(27.6)	ref	

*n (%): number of those willing to receive CA vaccination (the number of those willing to receive CA vaccination/ the total number of individuals in each category)

^#^n (%): number of those having positive attitude to CA vaccination (number of those having positive attitude to CA vaccination / the total number of individuals in each category)

In the multivariate analysis, among STD clinic attendees, those who had heard of CA demonstrated an over three-fold higher willingness to receive the HPV vaccine for CA (AOR = 3.14, 95% CI: 1.29–7.63, *p* = 0.0114). Additionally, the multivariate logistic regression model analysis indicated that STD clinic attendees who were aware of the relationship between HPV and CA (AOR = 2.56, 95% CI: 1.31-5.00, *p* = 0.0060), had heard of the HPV vaccines/ cervical cancer vaccines (AOR = 1.90, 95% CI: 1.02–3.54, *p* = 0.0444) and had proactively discussed CA or the vaccine with others (AOR = 1.95, 95% CI: 1.00-3.79, *p* = 0.0488) had better attitudes toward the HPV vaccine for CA.

### Healthcare costs for diagnostics and treatment for CA among STD clinic attendees and willingness to pay for the HPV vaccine for CA

**Fig. 2:**
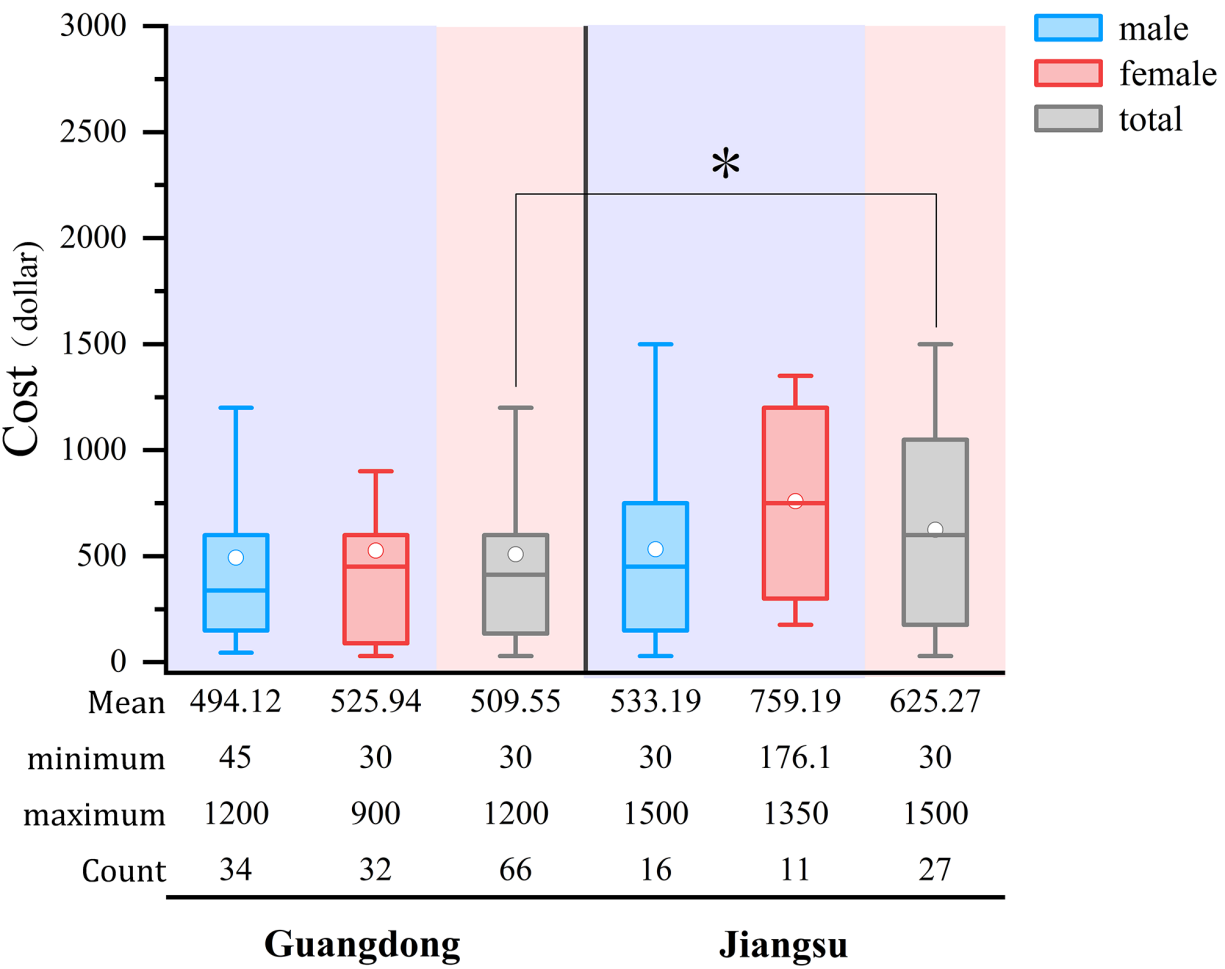
The costs for per visit of STD clinic attendees. *p < 0.05. The median lines in each box represents the average cost in each group; the lower and upper lines of the box represent the lower and upper values of 95% confidence interval of average cost; the lines beyond the boxes represents the range of cost in each group. The blue, red and grey boxes represent the group of females, male and overall STD clinic attendees respectively

A total of 93 STD clinic attendees with CA participated in the section on healthcare costs. Figure 2 showed the average cost for CA per visit according to different regions and sexes. The mean cost associated with diagnostics and treatment for CA per visit was $509.55 among STD clinic attendees from Guangdong, which was lower than the mean cost in Jiangsu ($625.27).

**Fig. 3:**
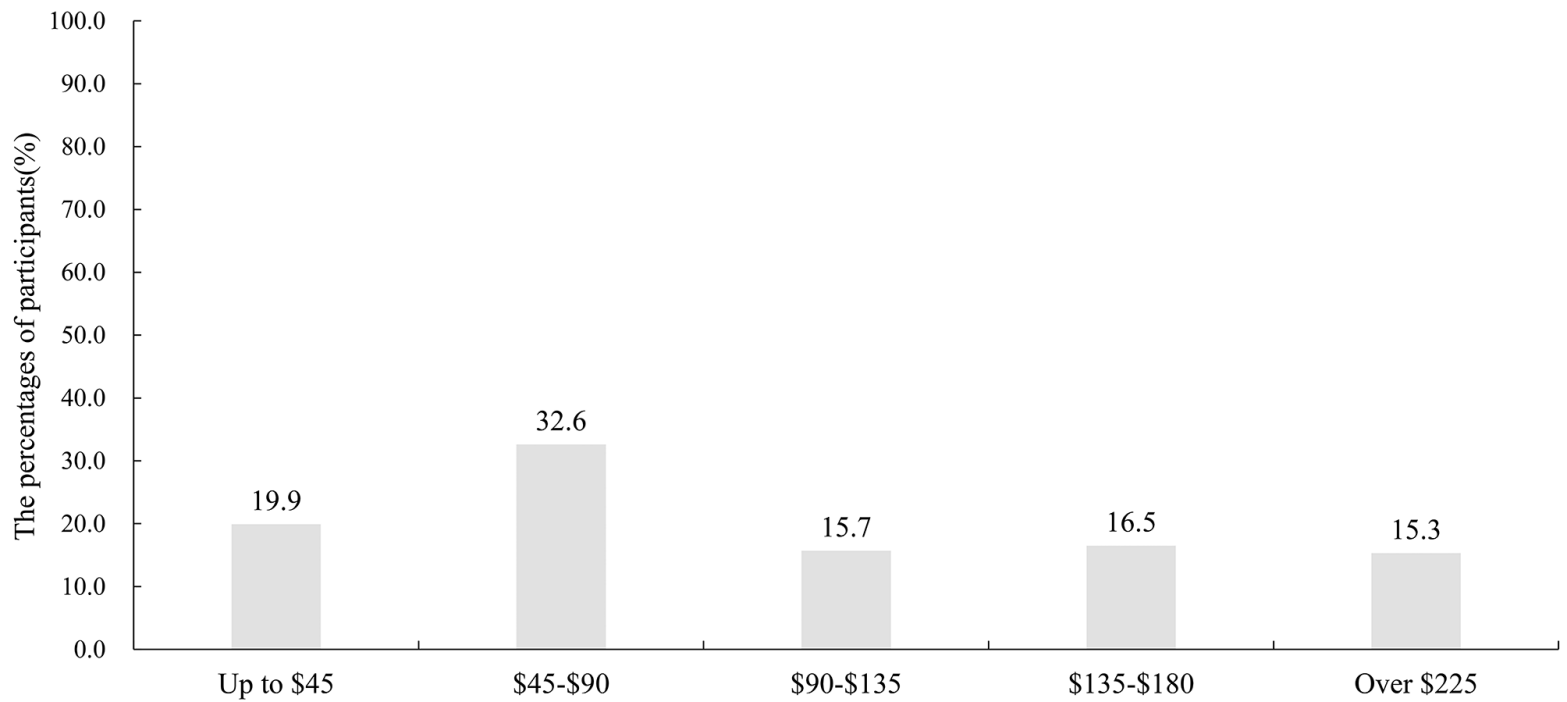
The acceptable range of payment for HPV vaccination. The x axis represents the acceptable range of payment for HPV vaccination. The y axis represents percentage of participants

Additionally, Fig. 3 revealed the acceptable payment ranges for HPV vaccination among participants, over half of the participants (52.5%) expected the vaccination to cost less than $90.

## Discussion

To the best of our knowledge, studies addressing how STD clinic attendees (both females and males) in China view the HPV vaccine for CA are rare. Here, we aimed to investigate willingness and attitude toward the HPV vaccine for CA among STD clinic attendees, and found that willingness and attitude toward the HPV vaccine for CA were positively associated with knowledge.

In our study, the HPV vaccine for CA willingness among STD clinic attendees in Guangdong and Jiangsu was high (85.8%,235/274), and participants were willing to adopt protective behaviors against CA. Under half of the STD clinic attendees (34.7%) had a positive attitude toward the HPV vaccine for CA. The results revealed that awareness of CA was significantly related to willingness to undergo the HPV vaccine for CA among STD clinic attendees. STD clinic attendees who were aware of the HPV vaccine and the relationship between HPV and CA, and had proactively discussed CA or the vaccine with others were more likely to have positive attitudes toward the HPV vaccine for CA. This finding implied that the knowledge of CA and related education was essential for vaccination willingness and positive attitudes, consistent with the findings of previous studies [17–19]. Although a relevant stable marital status and sexual behavior among participants were showed (55% of them were married and 80% of them had fixed sexual partners), the rate of individuals who had a history of CA was high (almost 50%) in our study, which might explain the observed high willingness towards vaccination.

Even among STD clinic attendees at high risk for CA, although most (72.3%) had heard of CA and 67.8%  had heard of HPV infection, 40.1% still were unaware of the connection between CA and HPV. The proportion of STD clinic attendees heard of HPV infection (67.8%) was higher than that found among university students in China (51.1%) [17], but lower than that of males recruited at a sexual health clinic in Italy (74.9%) [20], and significantly lower than that in MSM populations in Western countries (73–93%) [20–22]. Notably, although the participants were attendees of two STD clinics and a considerable proportion of them reported a history of CA (44.53%), the proportion of STD clinic attendees aware of the connection between CA and HPV (59.9%) was consistent with that of studies conducted on university students in China (54.0–67.5%) [17, 23, 24]. This result indicates that most STD clinic attendees have limited knowledge of the association between CA and HPV. The self-perception on knowledge of CA and HPV vaccines in STD clinic attendees was in a middle level. Other studies have shown that the more individuals know about HPV manifestations, signs, and forms of prevention, the better their health outcomes will be [25]. Educational programs can potentially broaden knowledge of the association between HPV and CA in the future, and improve knowledge of CA transmission routes, symptoms, risks, and treatments [26].

Our study data showed that patients with CA have a large financial burden of medical care, with the mean cost associated with diagnostics and treatment for CA per visit in Guangdong and Jiangsu being $509.55 and $625.27 respectively. A real-world cost analysis of England showed that lifetime costs per patient diagnosed with anogenital warts was equal to £872 (£884 and £856 for men and women, respectively) [27]. In Peru, given a population of 18.4 million adults between 18 and 60 years of age and a CA prevalence of 2.28%, the annual cost of treating CA was 25.1 million USD (uncertainty interval 16.9, 36.6) [28]. The cost per case of managing CA in Morocco, including recurrence, was estimated at €207–272 for women and €206–233 for men and the total annual cost of medical consultations for CA in Morocco ranged from €310,828 − 404,102 [29]. The annual incidence of CA in Mexico was estimated to be 547,200 cases, with an annual cost of $195 million USD, it also suggested that CA had a significant impact on public health [30].In our study, over half of STD clinic attendees expected the vaccination to cost less than $90.

Currently, all available HPV vaccines are only accessible to recommended-age females in China, so the affordability and accessibility of HPV vaccination are the priority challenges in the STD high-risk populations, especially among men. Therefore, another kind of HPV vaccine that targets solely HPV 6/11 for preventing CA, could play a role in addressing the issue of inadequate supplies and high price of current licensed HPV vaccines, to meet potential requirements of specific populations. Meanwhile, it should also be noted that an HPV-6/11 vaccine for CA may convey the message against STI, which might stigmatize and influence the acceptability of such a vaccine. It was encouraging that acceptance of the HPV vaccine for CA was high in our survey of STD clinic attendees and previous survey of MSM [31].

CA is a kind of benign lesion that is largely ignored among diseases caused by HPV infection (typically cancer); however, it could also lead to a decline in quality of life in the long term and has caused an unignorable disease burden in some populations. This study revealed that the willingness to be vaccinated and positive attitudes towards the HPV vaccine for CA are significantly linked to knowledge among STD clinic attendees, a very important group at high risk for STIs and HIV infection in China, to provide clues for future methods to address CA-associated health issues.

## Limitations

First, in this study, a preliminary exploration was conducted, but the deeper factors influencing the self-perception on knowledge of CA, acceptability of the HPV vaccine for CA and the impact of education on health were not considered. Second, although self-perception regarding knowledge of CA and HPV vaccines/ cervical cancer vaccines among STD clinic attendees was at a middle level, they were willing to accept the HPV vaccine for CA for a reasonable price. We think that an HPV-6/11 vaccine targeting CA might supplement existing HPV prevention strategies, potentially addressing the varied needs of different populations more effectively. However, more studies involving the implementation issues are warranted to be conducted, including but not limited to the cost-effectiveness analysis. Finally, the survey was conducted in 2017 (over 7 years ago) and the results of the survey may differ from the current situation, however, this difference may be very limited. For instance, the existing supply of several HPV vaccines remains in shortage; although the clinical guidelines for the treatment of CA in China in 2021 suggest that the quadrivalent and nonavalent vaccines containing HPV 6 and 11 are recommended for the prevention of CA, the current HPV vaccination strategy available in China until 2024 remains to prevent cervical cancer and not to recommend vaccination for the male population [32]. Over the past several years, people have been exhausted from coping with the COVID-19 pandemic, and the initiatives to pursue issues related to other infectious diseases were neglected and lacked (especially relatively benign lesions, such as CA). Therefore, although our study has some limitations in terms of timeliness, we believe that our results are valuable to CA prevention and control policy-makers in China.

## Conclusions

The willingness to undergo vaccination and attitudes toward the HPV vaccine for CA were positively associated with knowledge, and educational programs can have the potential to solve the barriers to the HPV vaccine for CA willingness and attitudes in the future, especially among individuals at high risk for STDs with negative attitudes.

## Data Availability

Authorization to access the data may be considered by the authors upon reasonable requests. Requests to access these datasets should be directed to the corresponding author, xuqi-ren@ntu.edu.cn.

## Abbreviations

HPV: Human papillomavirus
CA: Condyloma acuminata
STD: Sexually transmitted disease
STIs: Sexually transmitted infections
IQR: Interquartile range
OR: Crude odds ratio
AOR: Adjusted odds ratios
CI: Confidence intervals
LLMICs: low-income and lower-middle-income countries
